# Atypical Presentation of Ileal Actinomycosis Mimicking a Neuroendocrine Tumor: A Case Report

**DOI:** 10.7759/cureus.111568

**Published:** 2026-06-26

**Authors:** Ajith Vettuparambil, Sajith K Mohan, John Mathew Manipadam, Mahesh S, Ramesh Hariharan

**Affiliations:** 1 Gastrosurgery, Lakeshore Hospital and Research Centre, Cochin, IND

**Keywords:** actinomycosis, ileal diseases, ileum, intestinal obstruction, neuroendocrine tumors

## Abstract

We report a diagnostically challenging case of ileal actinomycosis in a 43-year-old woman presenting with features of subacute intestinal obstruction, radiologically and functionally mimicking a small bowel neuroendocrine tumor (NET). The patient’s presentation, imaging, and endoscopic findings initially raised a strong suspicion of malignancy, including differential consideration for NETs due to a mid-ileal stricture with an associated mesenteric mass, avid uptake on DOTATOC PET/CT, and mesenteric lymphadenopathy. However, the histopathology of the resected specimen confirmed an actinomycotic infection, highlighting the rare and deceptive nature of this chronic suppurative disease in the gastrointestinal tract.

## Introduction

Abdominal actinomycosis is a rare, chronic, suppurative, and infiltrative disease caused by Actinomyces israelii. This gram-positive, anaerobic filamentous bacterium resides commensally in the oropharynx, gastrointestinal tract, and female genital tract [1]. Despite its long historical recognition since the mid-19th century, the condition remains a diagnostic enigma, often earning the title of a "great mimicker" due to its ability to imitate cancers [2]. The ileocecal region is the most commonly affected intra-abdominal site, frequently arising after mucosal disruption from surgery, perforated appendicitis, or use of intrauterine devices [1,3]. Clinically, abdominal actinomycosis can present insidiously with nonspecific symptoms such as abdominal pain, weight loss, and palpable masses, frequently raising suspicion for intra-abdominal malignancies [4,5].

Imaging modalities typically reveal contrast-enhancing, infiltrative, and mass-like lesions with regional lymphadenopathy, prompting surgical intervention under the presumption of malignancy [1,4]. The diagnostic yield from preoperative biopsy is limited, with cultures frequently negative due to the organism's fastidious growth requirements. Histopathological examination revealing sulfur granules, filamentous bacteria, and suppurative granulomatous inflammation remains the gold standard for diagnosis [1,3]. Actinomycosis responds remarkably well to prolonged antibiotic therapy, usually penicillin-based, sometimes obviating the need for extensive surgical resections if diagnosed early [1,2].

We report a diagnostically challenging case of ileal actinomycosis in a middle-aged woman who presented with subacute intestinal obstruction and imaging findings suggestive of a small bowel neuroendocrine tumor (NET).

## Case presentation

A 43-year-old woman presented with intermittent colicky abdominal pain, recurrent episodes of bilious vomiting, abdominal distension, and loose stools for one month. Symptoms were associated with reduced oral intake and unintentional weight loss of approximately two kilograms over the preceding three months. There was no history of fever, gastrointestinal bleeding, night sweats, tuberculosis exposure, or prior inflammatory bowel disease. Her past medical history included a lower segment cesarean section 15 years ago. She had undergone medical management for uterine fibroids and two dilatation and curettage procedures, six and 13 years prior, for endometrial thickening and missed abortion, respectively. She had no other known comorbidities.

**Figure 1:**
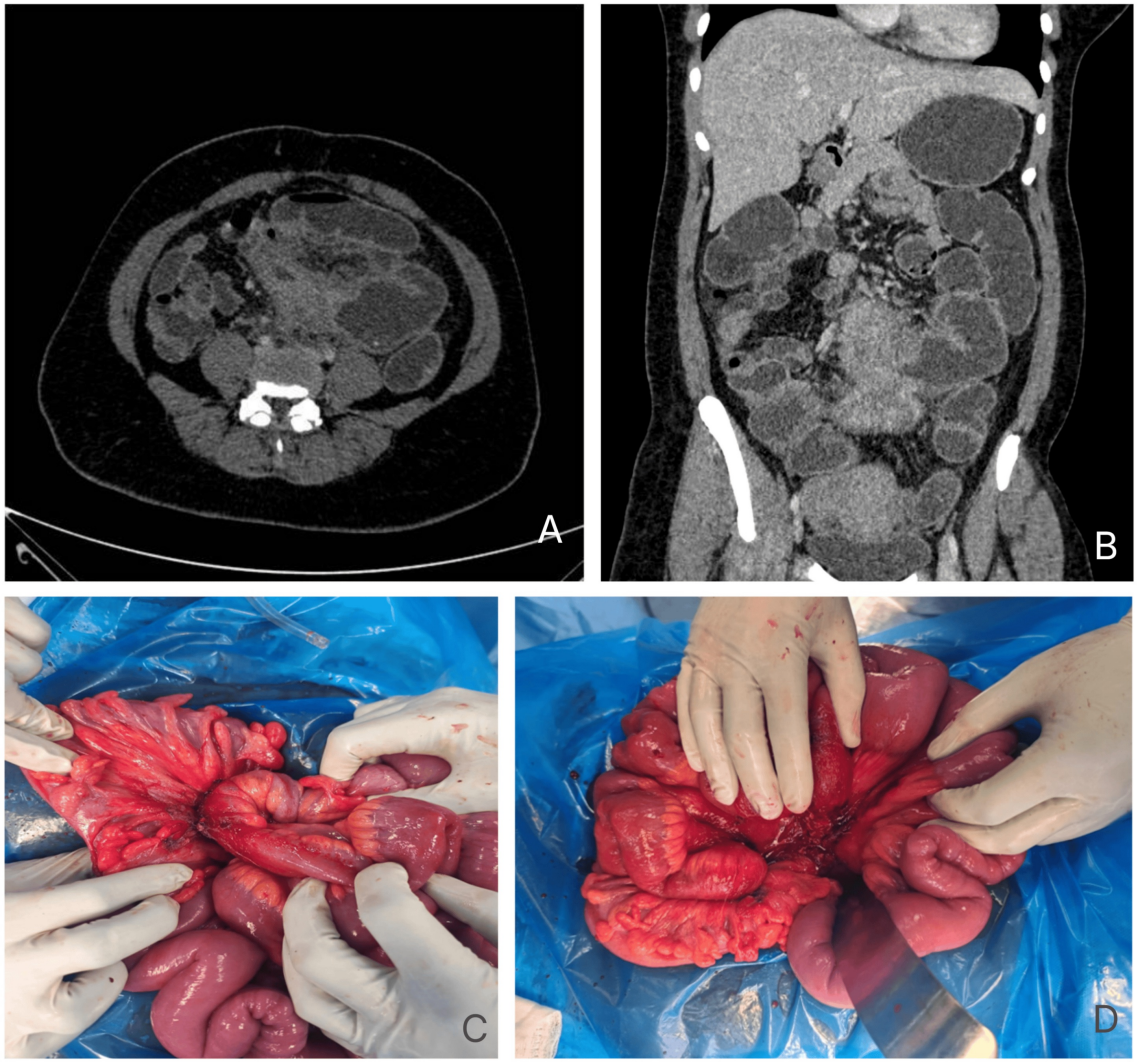
(A and B) CECT scan of the abdomen showed diffuse mural thickening involving a mid-ileal segment of approximately 6 cm, accompanied by luminal narrowing, surrounding inflammatory fat stranding, and an adjacent heterogeneously enhancing spiculated mass measuring 3.8 x 4.3 cm near the aortic bifurcation encasing the mesenteric root with prominent lymph nodes. (C and D) Terminal ileum was found to be densely adhered and clumped with the sigmoid colon and adjacent mesentery, forming a hard, ill-defined fibrotic mass at the umbilical level, just inferior to the aortic bifurcation CECT: Contrast-enhanced computed tomography

On examination, the patient was hemodynamically stable. Abdominal examination revealed mild distension and tenderness. No palpable abdominal mass, organomegaly, or free fluid was detected. Bowel sounds were exaggerated. Digital rectal examination was unremarkable. A contrast-enhanced computed tomography scan of the abdomen showed diffuse mural thickening involving a mid-ileal segment of approximately six centimeters, accompanied by luminal narrowing, surrounding inflammatory fat stranding, and an adjacent heterogeneously enhancing spiculated mass measuring 3.8 x 4.3 cm near the aortic bifurcation encasing the mesenteric root with prominent lymph nodes (Figure 1).

Laboratory investigations showed a haemoglobin level of 10.1 g/dL (reference range: 12.0-15.0 g/dL (adult female)) and a markedly elevated erythrocyte sedimentation rate of 100.3 mm/hr (reference range: 0-20 mm/hr (adult female)). Chromogranin A levels were 30.89 ng/mL (normal range: <78ng/mL). Antegrade enteroscopy performed the following day revealed circumferential edematous and erythematous mucosa in the proximal ileum with significant luminal narrowing and food stasis, consistent with stricture or infiltrative pathology. Scope advancement beyond this narrowed segment was not possible. A subsequent dye study confirmed a short segment of distal narrowing.

A whole-body ⁶⁸Ga-DOTATOC PET/CT scan was done to further evaluate for neuroendocrine pathology. It demonstrated DOTATOC avidity (SUV max: 8.3) in the mid-ileum, with a heterogeneously enhancing DOTATOC-avid soft tissue mass (36 × 24 × 64 mm; SUV max: 5.8). The difference in measurements reflects variations in CT and PET/CT imaging planes; both represented the same mesenteric lesion. Despite imaging findings suggestive of a NET, serum chromogranin A levels remained within the normal range, introducing diagnostic uncertainty and lowering the pre-test probability of a functional neuroendocrine neoplasm. Endoscopic biopsy from the proximal ileum revealed superficial ulceration, moderate ileitis, and nodular lymphoid hyperplasia, though no definitive evidence of malignancy was found.

Given the complexity and progressive symptoms, the patient underwent diagnostic laparoscopy followed by exploratory laparotomy. Intraoperatively, approximately 20 cm of the terminal ileum was found to be densely adhered and clumped with 20 cm of the sigmoid colon and adjacent mesentery, forming a hard, ill-defined fibrotic mass at the umbilical level, just inferior to the aortic bifurcation (Figure 1). Additionally, 20 cm of jejunum, located 150 cm distal to the duodenojejunal flexure, displayed multiple mucosal lesions with hard mesentery and proximal dilatation. All involved loops were densely adherent to the retroperitoneum, bilateral iliac vessels, and ureters. Minimal ascites was present. The patient underwent segmental jejunal resection, ileo-cecal resection, and sigmoid colon resection, including the mesentery involved. Restorative anastomoses were performed: side-to-side jejunojejunostomy, ileo-ascending colon anastomosis, and descending colorectal anastomosis.

**Figure 2:**
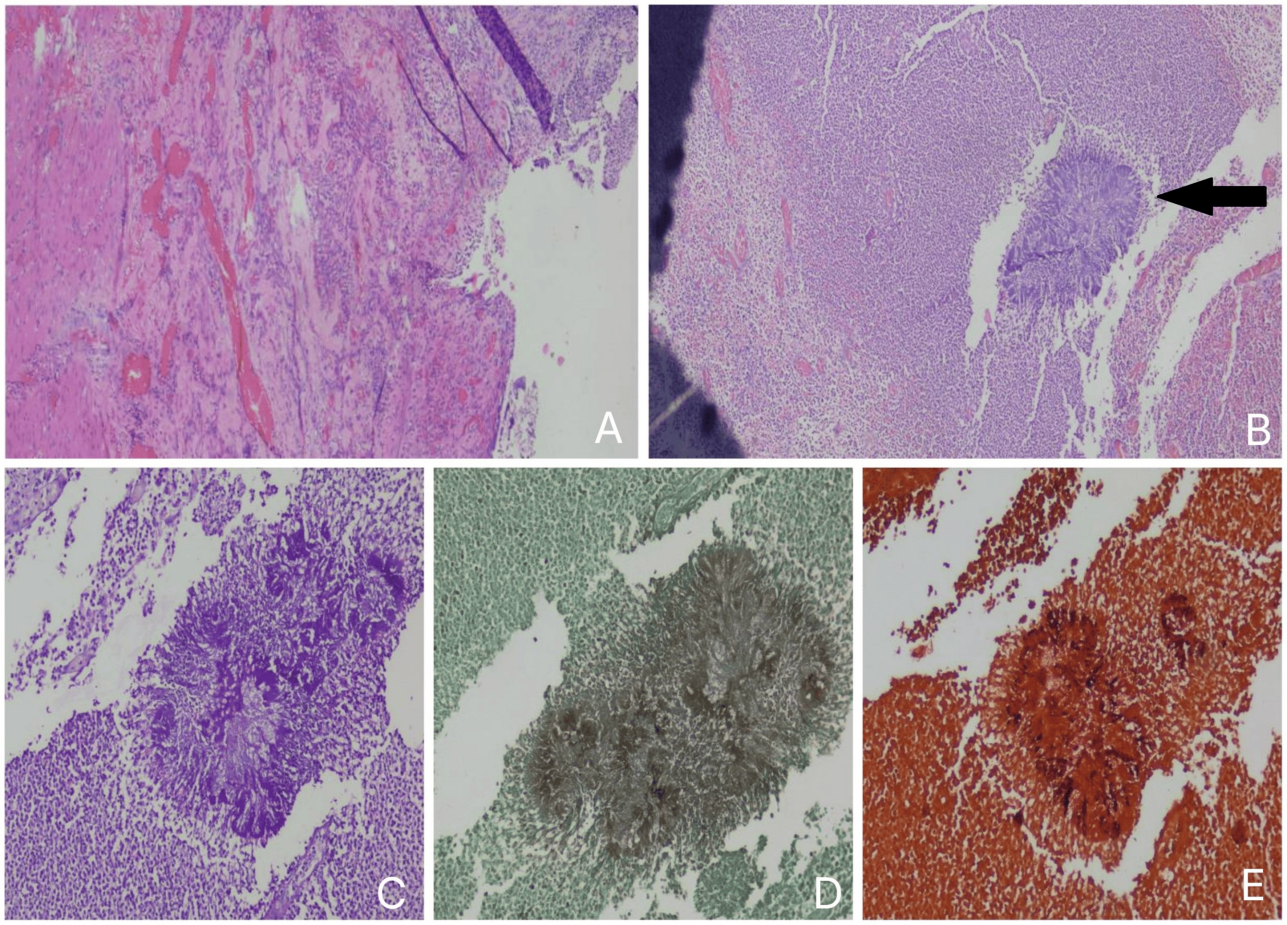
Histopathological examination of the resected specimen showing ileum with adhesion, dense inflammation, actinomycotic colonies, and serositis (A) Ileocecal mass with sigmoid colon. (B) Actinomycotic sulfur granule exhibiting the Splendore–Hoeppli phenomenon (black arrow). (C) PAS. (D) GMS. (E) Gram stain showing filamentous organisms consistent with Actinomyces species. PAS: Periodic Acid–Schiff stain; GMS: Grocott-Gomori Methenamine Silver stain.

Histopathological examination of the resected jejunum showed suppurative transmural inflammation with neutrophilic microabscesses, mesenteric serositis with reactive lymph nodes and characteristic sulfur granules surrounded by Splendore-Hoeppli material. The ileocecal and sigmoid colon specimens with the mesenteric mass demonstrated dense peri-ileal inflammation, neutrophilic microabscesses, and, most notably, actinomycotic colonies, a diagnostic hallmark of actinomycosis (Figure 2). Thirty lymph nodes showed reactive changes. Acid-fast bacilli staining, mycobacterial evaluation including tissue Gene Xpert MTB, and culture studies were negative, effectively excluding intestinal tuberculosis.

The final diagnosis was ileal actinomycosis with mesenteric involvement. The postoperative period was uneventful. The patient was treated with intravenous amoxicillin-clavulanate (Augmentin) 1.2 g every eight hours for three weeks, followed by oral Augmentin 625 mg three times daily for six months. At the 12-month follow-up, the patient remained asymptomatic, tolerated oral intake well, and showed no clinical or radiological evidence of recurrence.

## Discussion

Our case, involving a middle-aged woman with no overt immunosuppression, reinforces the disease's classic predilection for females. Although the patient had undergone previous pelvic procedures, the temporal relationship was remote and causality cannot be established. Nevertheless, prior mucosal disruption has been proposed as a potential predisposing factor in abdominal actinomycosis. This is consistent with patterns reported globally and in Indian cohorts, where many cases present with vague abdominal symptoms, mimicking tumors or carcinomatosis [6,7]. 

​In the most extensive systematic review to date, Manterola et al. analyzed 406 cases [8]. They confirmed that over 60% of abdominal actinomycosis cases present with tumor-like masses, with diagnoses typically made postoperatively​ [8]. This diagnostic challenge was echoed in our case, where radiological and functional imaging (DOTATOC PET/CT) strongly suggested a NET, a scenario also documented in other case reports where actinomycosis mimicked ileocecal, sigmoid or colonic malignancies​ [5,7,9]. Although DOTATOC PET/CT is highly sensitive for NETs, false-positive uptake may occur in inflammatory or infectious conditions due to SSTR-2 expression on activated immune cells.

​Therapeutically, actinomycosis is highly sensitive to prolonged high-dose penicillin therapy. Most guidelines recommend an initial course of intravenous antibiotics for 2-6 weeks, followed by oral therapy for 6-12 months​ [7-9,10]. Amoxicillin-clavulanate is an accepted alternative, particularly following complete surgical resection and in settings where prolonged outpatient therapy is required. Our patient demonstrated an excellent clinical response to this regimen.

From an Indian perspective, awareness of abdominal actinomycosis is still in its early stages of development. Cases remain underreported, partly due to a lack of suspicion and difficulty isolating Actinomyces species from cultures [7]. Histopathology, revealing sulfur granules and filamentous organisms, remains the diagnostic gold standard, as seen in our patient and the reviewed literature [6,11].

## Conclusions

Abdominal actinomycosis should be considered in the differential diagnosis of atypical abdominal masses or obstructive symptoms, particularly in patients with a history of prior pelvic or abdominal interventions. This case emphasizes the diagnostic challenge of ileal actinomycosis and its potential to mimic other pathologies. A high index of suspicion, supported by histopathological confirmation, can prevent unnecessary oncologic surgeries and enable curative antibiotic therapy. With appropriate surgical and antimicrobial management, the prognosis is excellent. Longer surveillance is required to confirm sustained disease control.
